# Preparation of Monolayer MoS_2_ Quantum Dots using Temporally Shaped Femtosecond Laser Ablation of Bulk MoS_2_ Targets in Water

**DOI:** 10.1038/s41598-017-10632-3

**Published:** 2017-09-11

**Authors:** Bo Li, Lan Jiang, Xin Li, Peng Ran, Pei Zuo, Andong Wang, Liangti Qu, Yang Zhao, Zhihua Cheng, Yongfeng Lu

**Affiliations:** 1 Laser Micro/Nano Fabrication Laboratory, School of Mechanical Engineering, Beijing Institute of Technology, Beijing, 100081 PR China; 2 Key Laboratory of Cluster Science, Ministry of Education, School of Chemistry, Beijing Institute of Technology, Beijing, 100081 PR China; 3 Department of Electrical and Computer Engineering, University of Nebraska-Lincoln, Lincoln, NE 68588-0511 USA

## Abstract

Zero-dimensional MoS_2_ quantum dots (QDs) possess distinct physical and chemical properties, which have garnered them considerable attention and facilitates their use in a broad range of applications. In this study, we prepared monolayer MoS_2_ QDs using temporally shaped femtosecond laser ablation of bulk MoS_2_ targets in water. The morphology, crystal structures, chemical, and optical properties of the MoS_2_ QDs were characterized by transmission electron microscopy, X-ray diffraction, Raman spectroscopy, X-ray photoelectron spectroscopy, UV–vis absorption spectra, and photoluminescence spectra. The analysis results show that highly pure, uniform, and monolayer MoS_2_ QDs can be successfully prepared. Moreover, by temporally shaping a conventional single pulse into a two-subpulse train, the production rate of MoS_2_ nanomaterials (including nanosheets, nanoparticles, and QDs) and the ratio of small size MoS_2_ QDs can be substantially improved. The underlying mechanism is a combination of multilevel photoexfoliation of monolayer MoS_2_ and water photoionization–enhanced light absorption. The as-prepared MoS_2_ QDs exhibit excellent electrocatalytic activity for hydrogen evolution reactions because of the abundant active edge sites, high specific surface area, and excellent electrical conductivity. Thus, this study provides a simple and green alternative strategy for the preparation of monolayer QDs of transition metal dichalcogenides or other layered materials.

## Introduction

Recently, graphene-like layered transition metal dichalcogenides (TMDs) have attracted substantial research attention because of their distinct physical and chemical properties^1, 2^. Molybdenum disulfide (MoS_2_), a typical TMD with a sizable intrinsic bandgap, comprises layered S–Mo–S sandwiched structures held by relatively weak Van der Waals forces; therefore, MoS_2_ can be readily cleaved along the layer surface^3, 4^. As a structure changes from a thin layer to a monolayer, MoS_2_ transforms from an indirect-bandgap semiconductor (1.2 eV for bulk material) to a direct-bandgap semiconductor (1.9 eV for monolayer)^4–6^. This unique structure and properties enable MoS_2_ to be applied in many fields, including biomedicine, energy storage, gas sensing, catalysis, and electronics engineering^7^. Monolayer MoS_2_ quantum dots (QDs), which possess strong quantum confinement, edge effects^8^, and direct-bandgap properties, are important MoS_2_-based nanostructures that have attracted considerable research attention in recent years. The abundant active edge sites, high specific surface area, and excellent electrical conductivity, together with the strong hydrogen adsorption properties^9–13^, have made monolayer QDs efficient electrocatalysts for hydrogen evolution reactions (HERs). Because the small size particles posses strong optical nonlinearity properties^14, 15^, the QDs are a promising photoluminescence (PL) material for biomedical and optical-imaging applications^16, 17^. In addition, the more uniform the size distribution of the monolayer QDs is, the more stable the quantum size confinement effects and the stronger the PL properties of the QDs are. Thus, there is tremendous demand to elucidate an effective method for preparing uniform-size monolayer MoS_2_ QDs.

To date, several strategies for preparing MoS_2_ QDs have been proposed, including sonication combined with solvothermal treatment synthesis^18^, lithium intercalation^19^, liquid exfoliation in organic solvents^20^, hydrothermal synthesis^21^, electrochemical etching^22^, electro-Fenton reaction processing^23^, and grinding exfoliation^9^. These methods all have their own advantages; for example, liquid exfoliation can provide a high yield of MoS_2_ QDs, chemical intercalation can yield 1T-phase MoS_2_ QDs with excellent HERs catalytic reactivity, and one-pot hydrothermal synthesis can yield heterostructures in a single step. However, all of these methods also have drawbacks: chemical intercalation may introduce metallic heteroatoms in MoS_2_, resulting in structural destruction and loss of the pristine semiconducting properties of MoS_2_; liquid exfoliation may be time consuming and requires the use of hazardous organic solvents; electrochemical etching may require harsh conditions, complex chemical reactions, and multiple steps as well as an intricate post-treatment process to remove byproducts; and grinding exfoliation may result in large size distributions and multilayer MoS_2_ nanoparticles. In short, the production of monolayer MoS_2_ QDs with uniform size distribution through a fast, simple, green, and one-step method remains a challenge.

Here, we propose the temporally shaped femtosecond laser ablation of bulk MoS_2_ targets in water as a novel approach for the fast, green, and one-step preparation of uniform monolayer MoS_2_ QDs. Femtosecond lasers with ultrahigh peak power densities (typically > 10^13^ W cm^−2^) and ultrashort pulse durations have unique characteristics, such as nonthermal effect and nonlinear nonequilibrium processing^24–26^, giving rise to the possibility of monolayer photoexfoliation of two-dimensional materials^27, 28^. Furthermore, through temporal shaping of a conventional single pulse into a two-subpulse train, we can improve the production rate of MoS_2_ nanomaterials (including nanosheets, nanoparticles, and QDs) and the ratio of uniform, small-size MoS_2_ QDs. The underlying mechanism is a combination of multilevel photoexfoliation of monolayer MoS_2_ and water photoionization–enhanced light absorption; this mechanism can be induced and well controlled using temporally shaped femtosecond lasers. The proposed method has the following major advantages over current strategies: (1) the preparation time is considerably reduced; (2) the method is environmentally-friendly; the bulk MoS_2_ targets are ablated in water; hence, high-purity MoS_2_ QDs can be obtained without the use of metallic heteroatoms or chemical reagents. Thus, the as-prepared MoS_2_ QDs can be directly used for further characterization and applications without intricate post-treatment processing. In particular, the use of the temporally shaped femtosecond laser has the following advantages: (1) uniform monolayer MoS_2_ QDs with a size distribution of 1–5 nm can be prepared; (2) small monolayer MoS_2_ QDs can be prepared in high yield (36.73 wt%); these QDs have numerous active edges and excellent electrocatalytic activity for HERs (low onset potential of approximately 140 mV, small Tafel slope of approximately 66 mV dec^−1^). Moreover, the temporally shaped femtosecond laser has the potential to be a fast, uniform, green, and alternative strategy to facilely and efficiently prepare QDs of TMDs or other layered materials for abroad applications in biomedical, optical imaging, energy storage, and catalysis areas.

## Results and Discussion

**Figure 1:**
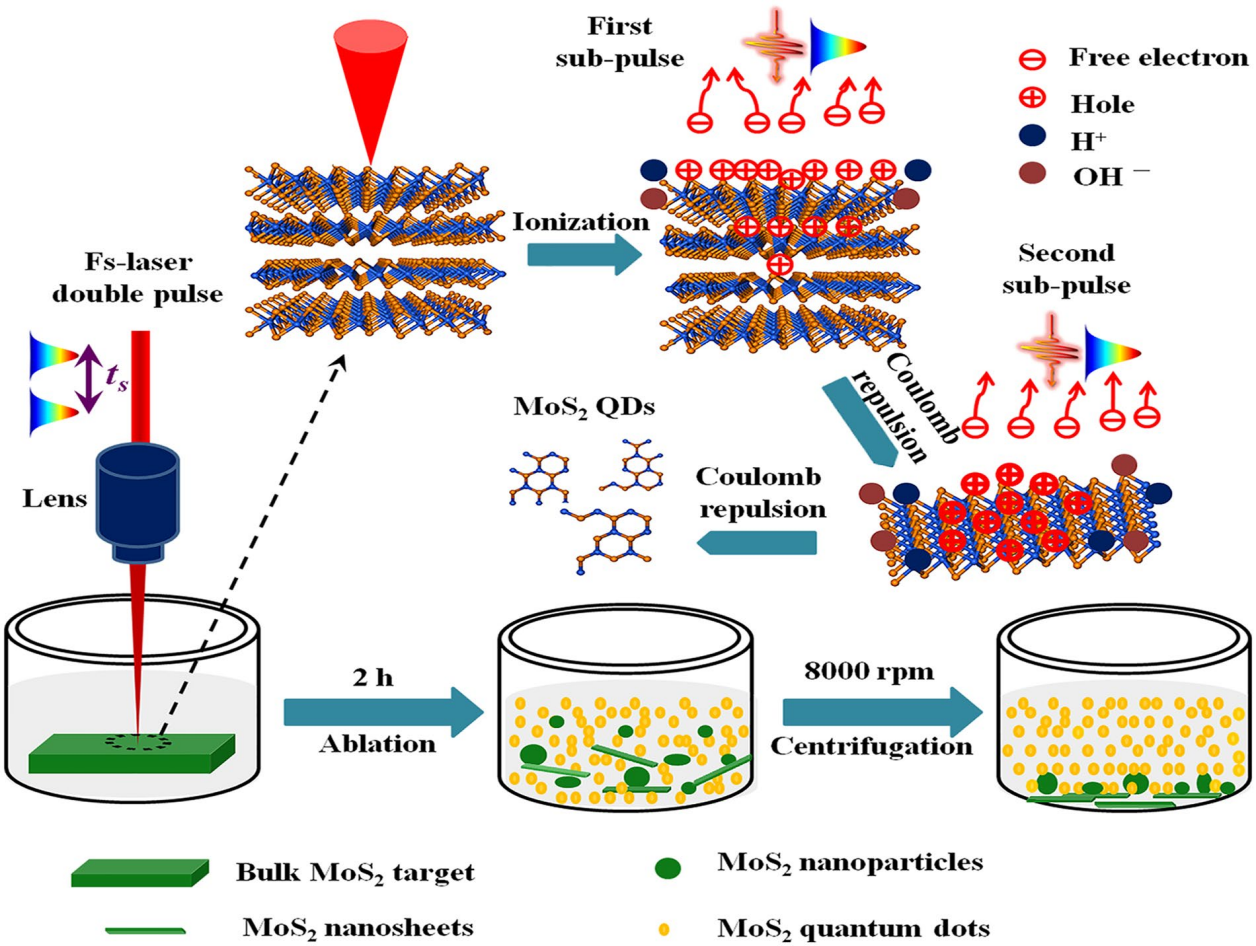
Schematic representation of the process mechanism for temporally shaped femtosecond laser two-subpulse train ablation of bulk MoS_2_ targets in water.

A novel fast, green, and one-step method was proposed to prepare high-purity, uniform, and small (size distribution of 1–5 nm) monolayer MoS_2_ QDs through temporally shaped femtosecond laser ablation of bulk MoS_2_ targets in water. The experimental setup for the temporally shaped femtosecond laser ablation is shown in Supplementary Figure S1, wherein a conventional femtosecond pulse is temporally shaped into two subpulses (energy ratio 1:1, total laser fluence 0.77 J cm^−2^) with time delays ranging from 0 to 10 ps. The underlying mechanism is a combination of multilevel photoexfoliation of monolayer MoS_2_ and water photoionization–enhanced light absorption; this mechanism can be induced and well controlled using a temporally shaped femtosecond laser, as shown in Fig. 1. As the first subpulse (with fluence slightly higher than the ablation threshold of 0.24 J cm^−2^ for bulk MoS_2_)^29^ irradiates on the MoS_2_ surface, the heated electrons spill out. Therefore, the MoS_2_ interlayer interactions are reduced by Coulomb repulsion^28^, which can trigger first-level photoexfoliation (detachment of the multilayer MoS_2_ QDs/nanosheets from bulk materials) within the femtosecond time scale. The electron and hole recombination in the MoS_2_ surface lasts from 2 to 100 ps after the first subpulse^30^. As the second subpulse with 10 ps time delay irradiates, the enhanced Coulomb repulsion due to enhanced ionization–induced charge accumulation results in the MoS_2_ QDs/nanosheets or bulk MoS_2_ surface achieving second-level photoexfoliation (small monolayer MoS_2_ QDs preparation). Meanwhile, the first subpulse can ionize the water molecules within the hydrogen-bond associated time from 200 fs to 13 ps^31^. The increase in electron density due to the ionization of water molecules via the first subpulse enhances the light absorption for the second subpulse, which is responsible for the high yield of MoS_2_ QDs. While in the case of femtosecond laser single pulse ablation of bulk MoS_2_ targets in water, the laser pulse has a total fluence of 0.77 J cm^−2^, which is much greater than the ablation threshold of bulk MoS_2_. Therefore, when the femtosecond laser single pulse irradiates on the MoS_2_ surface, it can induce a localized transient free-electron density that is much higher than the critical density. This leads to thermal phase-change mechanisms (*e.g*. melting and evaporation) dominating the ablation process, thereby resulting in more large size nanosheets/nanoparticles and a wide size distribution. In short, through temporally shaping of a conventional single pulse into a two-subpulse train, we can improve the production rate of MoS_2_ nanomaterials (including nanosheets, nanoparticles, and QDs) and the ratio of uniform, small-size MoS_2_ QDs, a finding which corroborates previous reported results^32–34^.

**Figure 2:**
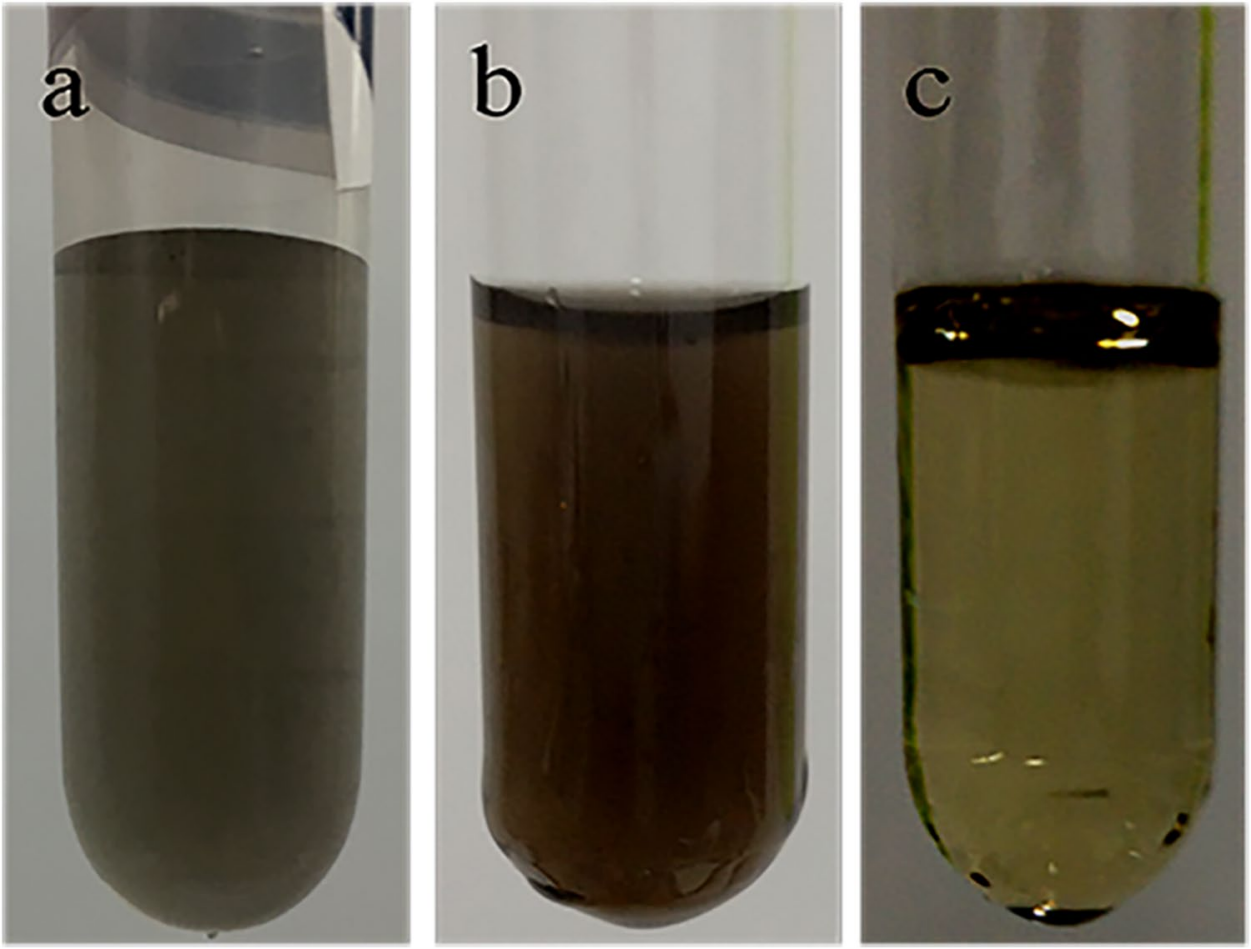
Photographs of the MoS_2_ aqueous solutions prepared using a femtosecond laser single pulse (**a**) before centrifugation, and temporally shaped femtosecond laser two-subpulse train (**b**) before and (**c**) after centrifugation.

Details about the synthesis and experimental setup of this study are reported in the Experimental Section. Scanning electron microscope (SEM) images of the surface of the original bulk MoS_2_ targets before and after temporally shaped femtosecond laser two-subpulse train ablation in water are shown in Supplementary Figure S2. After ablation, the dark-brown MoS_2_ aqueous solution with concentration of 166.02 μg/mL (Fig. 2b) was allowed to settle for 2–4 hours, following which it was centrifuged for 10 minutes at 8000 rpm to obtain a transparent light-yellow supernatant solution with concentration of 60.98 μg/mL (Fig. 2c). The yield of MoS_2_ QDs—defined as the concentration of the transparent light-yellow supernatant solution that mainly consists of QDs obtained after concentration relative to the concentration of the dark-brown MoS_2_ aqueous solution comprising nanosheets, nanoparticles, and QDs before concentration—was approximately 36.73 wt%. For comparison, a photograph of the MoS_2_ aqueous solution prepared using a femtosecond laser single pulse before centrifugation is shown in Fig. 2a. The concentration of this MoS_2_ aqueous solution with concentration of 79.06 μg/mL was obviously smaller than that of the solution prepared through temporally shaped femtosecond laser two-subpulse train ablation. The ablation time was 2 hours and the production rate for femtosecond laser single pulse and temporally shaped pulse train was 3.29 and 6.92 μg/min, respectively. Thus, we improved the production rate of MoS_2_ nanomaterials (including nanosheets, nanoparticles, and QDs) by 2.1 times through temporally shaping a conventional single pulse into a two-subpulse train.

**Figure 3:**
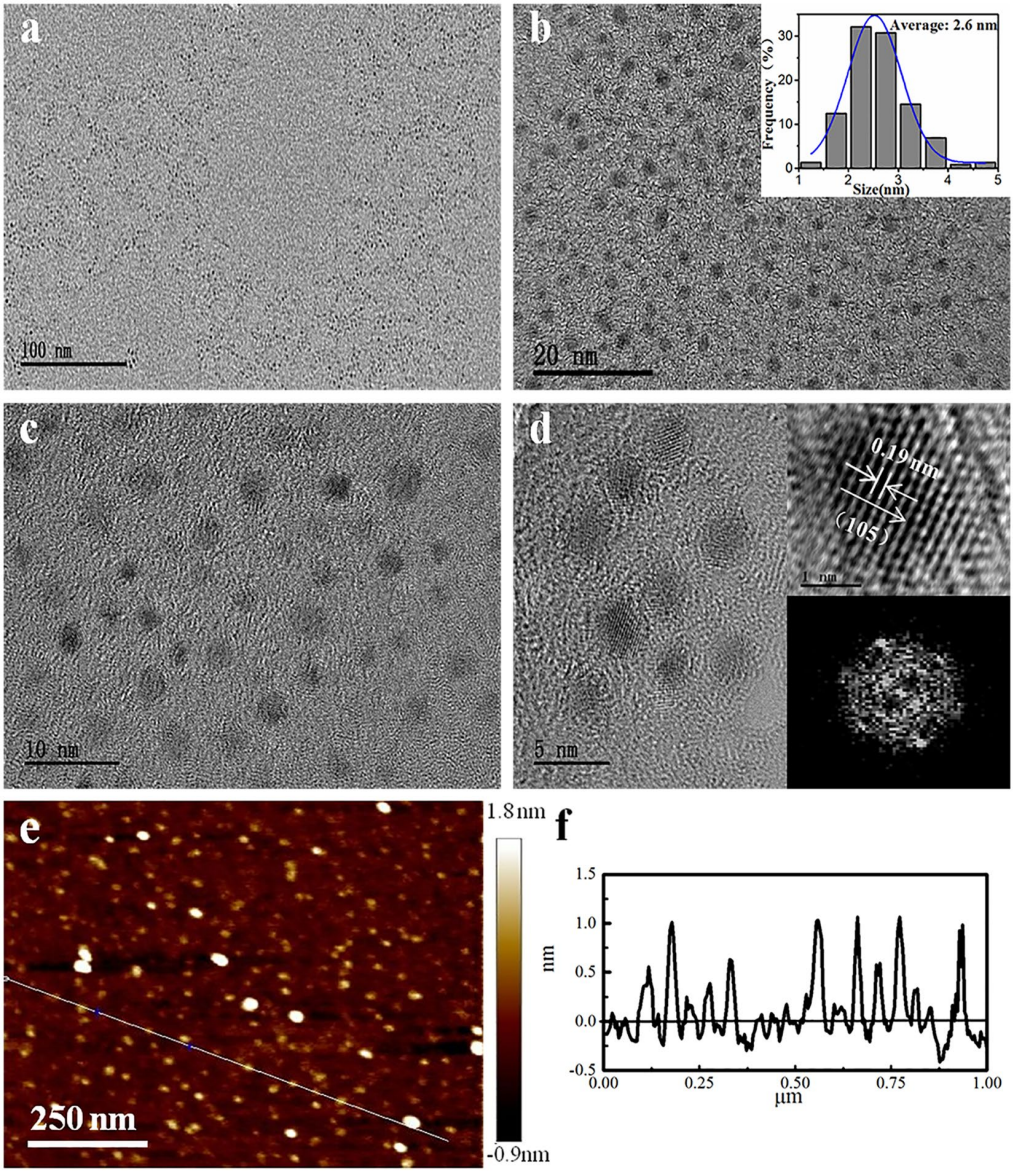
TEM images (**a–d**) at different scale bars: (**a**) 100 nm, (**b**) 20 nm, (**c**) 10 nm, and (**d**) 5 nm, and AFM image (**e**) 250 nm. Inset image in (**b**) shows the size distribution. Inset images in (**d**) show the HRTEM images (upper panel) and FFT pattern (lower panel) of the crystal of MoS_2_ QDs. (**f**) Height profile corresponding with the line in (**e**).

Transmission electron microscopy (TEM) was used to characterize the size, morphology, and lattice structure of the as-prepared MoS_2_ QDs. Figure 3a reveals that the as-prepared MoS_2_ QDs obtained after centrifugation were homogeneously dispersed, with little aggregation. Figure 3c and d show that the as-prepared MoS_2_ QDs were monodispersed crystalline. Additionally, the inset of Fig. 3b shows the size distribution of the as-prepared MoS_2_ QDs, exhibiting a relatively narrow size distribution of approximately 1–5 nm and an average lateral dimension of approximately 2.6 nm. Before centrifugation, however, the nanomaterials prepared through femtosecond laser single-pulse ablation exhibited a broad distribution of approximately 1–120 nm and an average lateral dimension of approximately 36.3 nm; additionally, the ratio of the QDs that were smaller than 10 nm was only 16.7% (Supplementary Figure S3a). By contrast, the ratio of the small QDs prepared using the temporally shaped femtosecond laser two-subpulse train, and which were variously sized from approximately 1–10 nm, was more than 50% (Supplementary Figure S3(b–d)). These results revealed that temporally shaping a conventional single pulse into a two-subpulse train and then optimizing the delay between the two ultrashort subpulses greatly improves the ratio of uniform small-size MoS_2_ QDs. This improvement is due to the multilevel photoexfoliation of MoS_2_ that is triggered by an increase in the localized transient free-electron density to slightly higher than the critical density, and then foster the nonthermal phase-change mechanism (Coulomb repulsion)^28^ to dominate the ablation process, resulting in a high photon absorption efficiency and narrow size distribution^25, 35^. In the case of femtosecond laser single pulse with the same total energy, a localized transient free-electron density that is much higher than critical density can be induced; this leads to the thermal phase-change mechanisms (*e.g*. melting and evaporation) dominating the ablation process and thereby results in more large-size nanosheets/nanoparticles and a broad distribution^36, 37^. The high-resolution TEM (HRTEM) image in the inset of Fig. 3d clearly shows that the as-prepared MoS_2_ QDs have parallel and ordered lattice fringes, indicating that the QDs are well crystallized. The d-spacing of the as-prepared MoS_2_ QDs is 0.19 nm, which is assigned to the (105) face of the MoS_2_ crystal. The corresponding fast Fourier transform (FFT) pattern similarly revealed the hexagonal lattice structure of the as-prepared MoS_2_ QDs. Atomic force microscopy (AFM) measurements were also performed to confirm the morphology and thickness of the as-prepared MoS_2_ QDs. As shown in the AFM height profile (Fig. 3f), the thickness of most of the QDs was less than 1 nm, indicating that most of the as-prepared MoS_2_ QDs were monolayer, in accordance with previous reported results^18, 21, 38, 39^.

To confirm that the temporally shaped femtosecond laser method has universality (*i.e*., can produce QDs of other TMDs and other layered materials), WS_2_ QDs and graphene QDs (GQDs) were also prepared using this method, as shown in Supplementary Figures S4 and S5. After 2 hours of femtosecond laser two-subpulse train ablation of the bulk WS_2_ targets in water and then 10 minutes of centrifugation at 8000 rpm, we obtained WS_2_ QDs that were homogeneously dispersed with little aggregation (Supplementary Figure S4a) and highly crystalline (Supplementary Figure S4c). The inset of Supplementary Figure S4b shows the size distribution of the as-prepared WS_2_ QDs, and indicates that they had a relatively narrow size distribution that varied from approximately 1–8 nm and an average lateral dimension of approximately 4.1 nm. Similarly, we have also preliminary attempted to prepare GQDs by temporally shaped femtosecond laser ablation of graphene oxide (GO) dispersions. After 1 hour of femtosecond laser two-subpulse train ablation, we obtained GQDs that were homogeneously dispersed with little aggregation and were almost smaller than 5 nm (Supplementary Figure S5). However, this was a preliminary experiment, and additional research should be conducted to verify the ability of our method to prepare GQDs.

**Figure 4:**
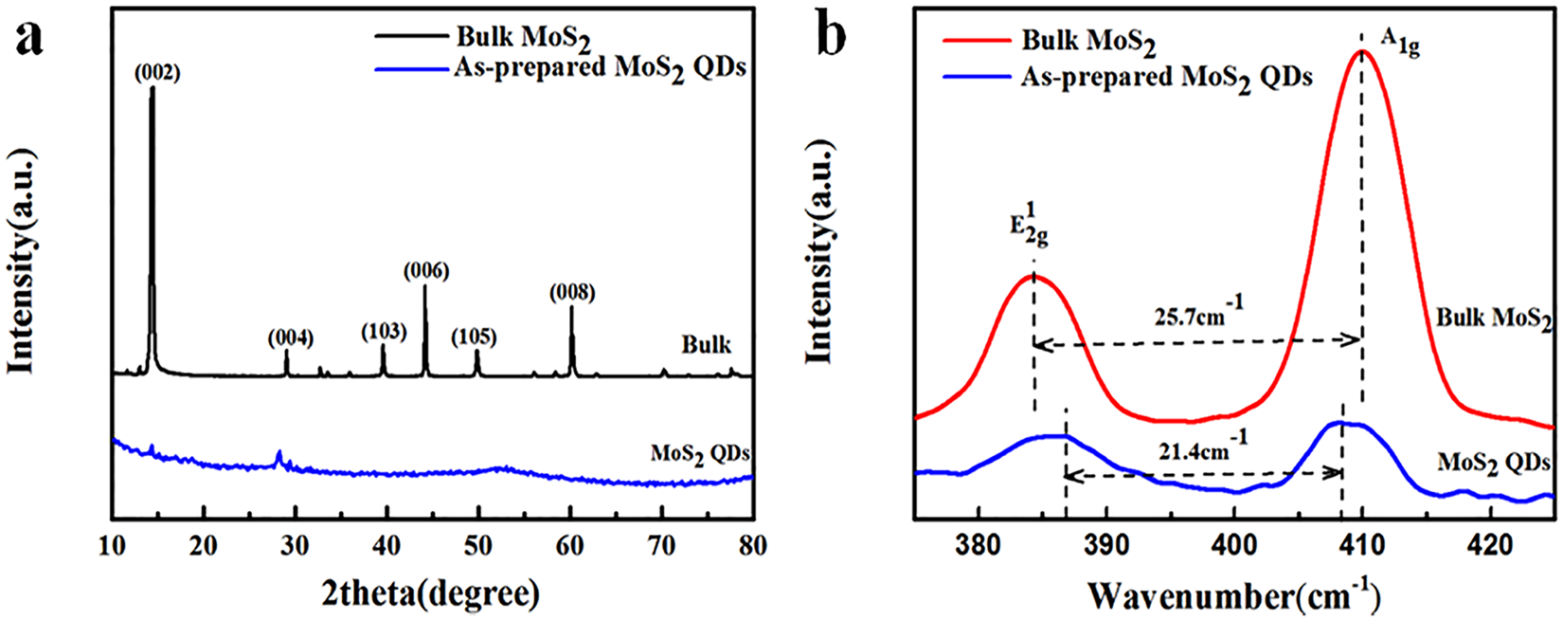
(**a**) XRD pattern and (**b**) Raman spectra of bulk MoS_2_ and as-prepared MoS_2_ QDs.

The crystal structure of the as-prepared MoS_2_ QDs was systematically characterized through X-ray diffraction (XRD) and Raman spectroscopy. As shown in Fig. 4a, the original bulk MoS_2_ target had an obviously strong diffraction peak at 2θ = 14.4° corresponding to the (002) face, indicating that the original materials had a multilayer structure. In addition, several lower peaks located at 2θ = 29°, 2θ = 39.6°, 2θ = 44.2°, 2θ = 49.8°, and 2θ = 60.2° can be observed, which are assigned to the (004), (103), (006), (105), and (008) faces, respectively (JCPDF (37–1492)). If the materials are thin or monolayer, no signals or peaks exist on the XRD patterns because there is no constructive interference from the aligned crystal planes^18^. After femtosecond laser ablation, the (002) peak of the as-prepared MoS_2_ QDs almost disappeared, confirming that the QDs were thinned to monolayer or few layers, which was in accordance with the AFM results. Further, the small peak at 2θ = 28.4°, corresponding to the (004) face, probably resulted from the partial restacking of QDs during the drying process^18, 40^.

Raman spectra are a common and facile measurement to investigate the layers of MoS_2_ materials. In Fig. 4b, the bulk MoS_2_ target shows two characteristic Raman peaks at 384.3 and 410 cm^−1^, corresponding to E_2g_^1^ (in-plane optical vibration of the Mo−S bond in opposite directions) and A_1g_ (out-of-plane optical vibration of S atoms) active modes, respectively. By contrast, the A_1g_ and E_2g_^1^ modes of the as-prepared MoS_2_ QDs had a red-shift of 1.8 cm^−1^ and blue-shift of 2.5 cm^−1^, respectively. In other words, the frequency difference between the A_1g_ and E_2g_^1^ modes reduced from 25.7 cm^−1^ for bulk MoS_2_ to 21.4 cm^−1^ for QDs, indicating that the as-prepared MoS_2_ QDs were thinned to mono- or bi-layers, which was consistent with previous reports^41^. As shown in Supplementary Figure S4d, the bulk WS_2_ also shows two characteristic Raman peaks at 351.0 and 420.6 cm^−1^ corresponding to E_2g_ and A_1g_ active modes, respectively. By contrast, the E_2g_ and A_1g_ active modes of the as-prepared WS_2_ QDs were at 353.0 and 420.4 cm^−1^ and had a blue-shift of 2 cm^−1^ and red-shift of 0.2 cm^−1^, respectively. These results indicated that the thickness of the as-prepared WS_2_ QDs was thinned to a few layers.

**Figure 5:**
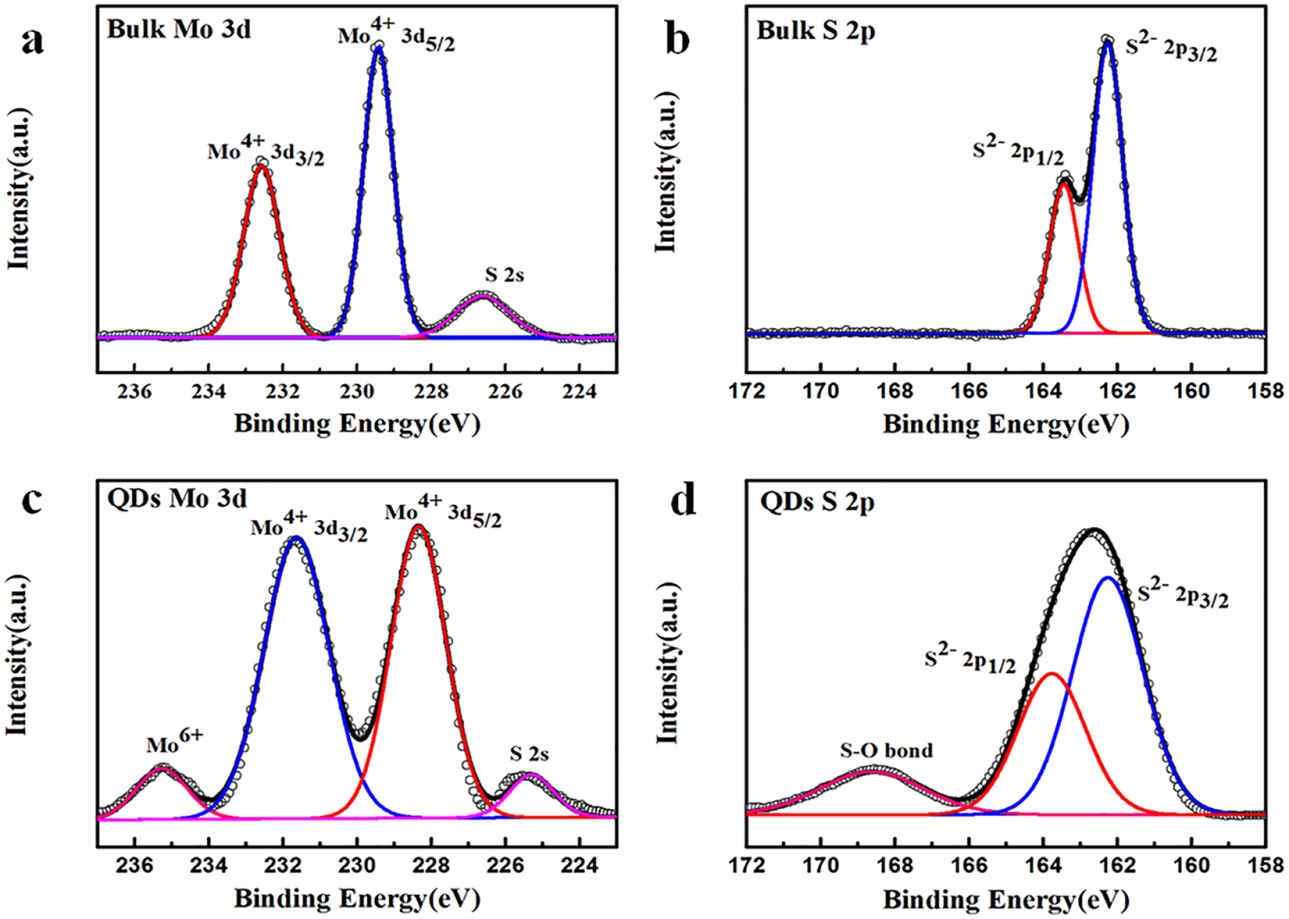
High-resolution XPS of Mo 3d and S 2p spectra for (**a**) and (**b**) bulk MoS_2_; (**c**) and (**d**) the as-prepared MoS_2_ QDs.

X-ray photoelectron spectroscopy (XPS) measurements were performed to investigate the chemical composition and phase state of the as-prepared MoS_2_ QDs. The high-resolution XPS spectra of original bulk MoS_2_ (Fig. 5a) exhibits two obvious peaks at 229.4 and 232.6 eV, which belong to the Mo^4+^3d5/2 and Mo^4+^3d3/2 components of 2H-MoS_2_, respectively. Another small peak at 226.6 eV is the S 2 s of MoS_2_ and other peaks observed at 162.3 and 163.5 eV are assigned to S^2−^2p3/2 and S^2−^2p1/2 in 2H-MoS_2_, respectively (Fig. 5b). The Binding energies of Mo 3d and S 2p regions reveal that the original bulk MoS_2_ has a strong trigonal prismatic structure. In the Mo 3d spectra of the as-prepared MoS_2_ QDs, the Mo^4+^ doublet and S 2 s peak were observed at 231.6, 228.3, and 225.3 eV (Fig. 5c). In addition, a new small peak located at a higher binding energy (235.3 eV) is ascribed to Mo^6+^, demonstrating that the Mo edges in MoS_2_ QDs are slightly oxidated during the transition from the Mo^4+^ state to the Mo^6+^ state. Similarly, the S 2p spectra shows the S^2−^ doublet at 162.3 and 163.7 eV (Fig. 5d). Further, a peak at a higher binding energy (168.6 eV) is observed, which corresponds to the presence of an S–O bond^42^, indicating partial oxidation of the S edges in MoS_2_ QDs. The oxidation percentage of Mo and S edges were 6.6% and 14.5%, respectively. This slight oxidation can be attributed to femtosecond laser ablation process, in which the femtosecond laser–induced transient high temperatures at the centre of the focal point focused on the MoS_2_–water interface. Although such oxidation can somewhat modify the electronic properties of the original 2H-MoS_2_^12, 43^, this can also generate more active surface sites with high catalytic properties, thus improving the HERs^22, 44^. The full spectrum of the XPS survey of the as-prepared MoS_2_ QDs is shown in Supplementary Figure S6. Here, except for the XPS peaks of Si that come from the substrate, there are only C, O, Mo, and S elements in the as-prepared MoS_2_ QDs. These results demonstrate that the MoS_2_ QDs prepared through the temporally shaped femtosecond laser ablation of bulk MoS_2_ targets in water exhibited high purity, with no metallic heteroatoms or chemical reagents. Thus, they can be directly used for further characterization and applications.

**Figure 6:**
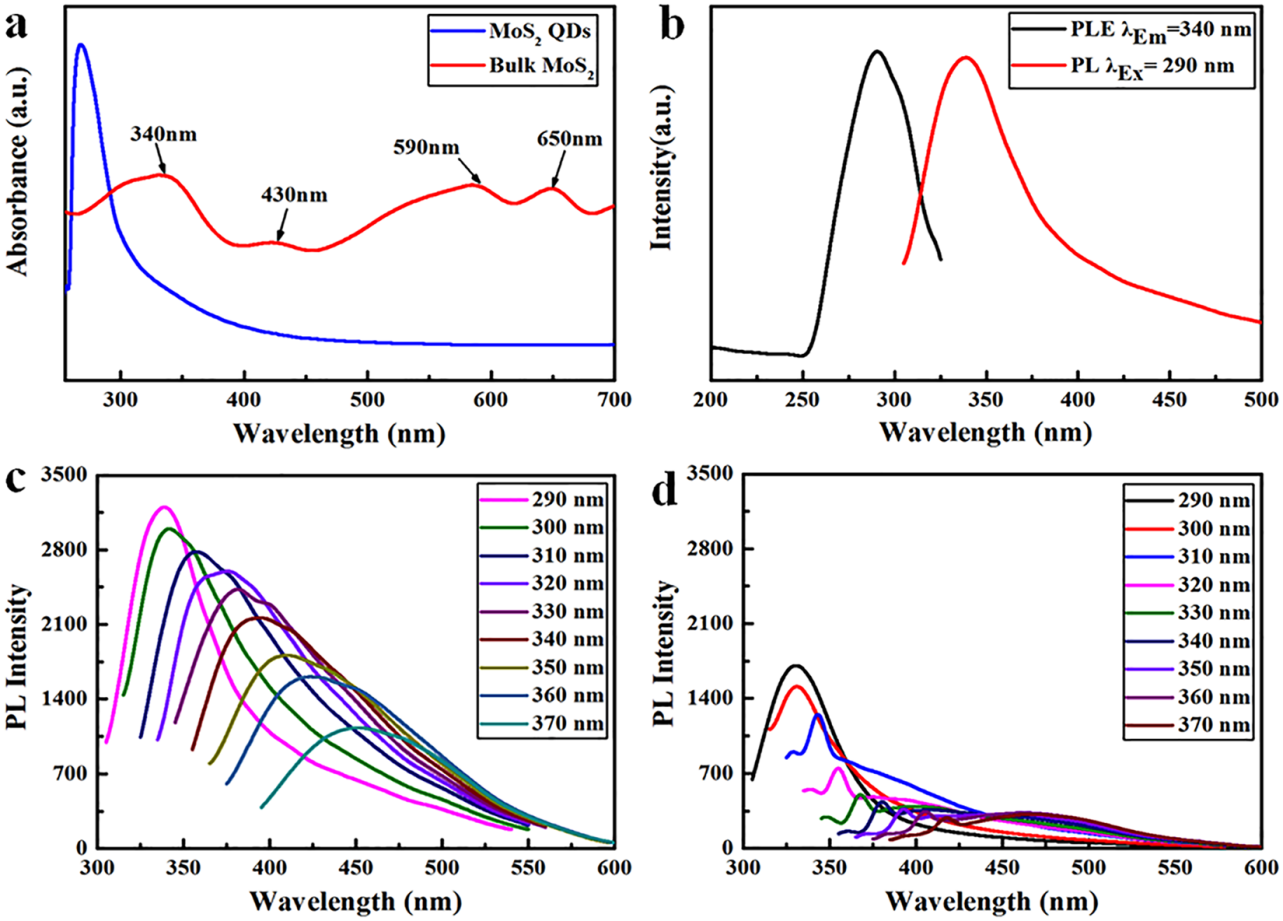
(**a**) UV–vis spectra of the bulk MoS_2_ and as-prepared MoS_2_ QDs. (**b**) Excitation and emission PL spectra of the as-prepared MoS_2_ QDs prepared in NMP. (**c**) Emission PL spectra of the as-prepared MoS_2_ QDs under different excitation wavelengths prepared in NMP. (**d**) Emission PL spectra of the NMP aqueous solution without MoS_2_ materials.

To further investigate the optical properties of the as-prepared MoS_2_ QDs, UV–vis absorption spectra were investigated (Fig. 6a). The peaks appearing at 340, 430, 590, and 650 nm are the characteristic absorption bands of the original bulk MoS_2_. The obvious peaks at 590 and 650 nm were assigned to the K point of the Brillouin zone, and the peaks at 340 and 430 nm were attributed to the direct transition from the deep valence band to the conduction band^18, 20, 45^. However, these four characteristic absorption bands disappear in the spectra of the as-prepared MoS_2_ QDs. Only one peak is observed in the near-UV region (λ < 300 nm), which was attributed to the excitonic features of MoS_2_ QDs^46^. Moreover, the strong blue-shift during optical absorption of the as-prepared MoS_2_ QDs was attributed to the quantum confinement and edge effects^11^ when the lateral size of the MoS_2_ QDs is reduced to <50 nm. According to the TEM results, the majority of the as-prepared MoS_2_ QDs are in the range of 1–5 nm and a strong blue shift is observed; this is similar to findings reported elsewhere^18, 21, 39, 47, 48^.

Apart from in water, we explored the temporally shaped femtosecond laser ablation of bulk MoS_2_ targets in other aqueous media, such as N-methyl pyrrolidone (NMP). The PL spectra of the MoS_2_ QDs prepared in NMP exhibit a strong emission peak at 340 nm under an excitation wavelength of 290 nm (Fig. 6b). The origin of PL from the present MoS_2_ QDs may be because of the exciton recombination at the electron (hole) trap constituted by the uncompensated positive (negative) charge at the dangling bonds^22^. In addition, the PL spectra of the as-prepared MoS_2_ QDs suspensions were measured at various excitation wavelengths. With an increase in the excitation wavelength from 290 to 370 nm, the PL emission peaks shift to longer wavelengths, namely from 340 to 450 nm (Fig. 6c). This phenomenon of excitation-dependent PL property confirmed the polydispersity of the as-prepared MoS_2_ QDs. This phenomenon may also be due to the presence of several trap states, as observed in GQDs^48–50^. Moreover, we characterized the emission PL spectra of the NMP aqueous solution without MoS_2_ materials (Fig. 6d). The PL intensity of all the emission peaks of the NMP solution is much lower than that of the as-prepared MoS_2_ QDs. In addition, the shape and position of the PL emission peaks of the NMP solution are visibly different from those of the as-prepared MoS_2_ QDs. Furthermore, to determine whether the NMP solution can be transformed into carbon dots by temporally shaped femtosecond laser ablation and then enhance the PL intensity of the MoS_2_, we performed the same experiments in the NMP solution without MoS_2_ materials. According to the resulting Raman spectra, no obvious Raman peaks are assigned to the carbon materials; this indicates that no carbon dots were decomposed to enhance the PL intensity of the MoS_2_ QDs (Supplementary Figure S7). Therefore, we suppose that femtosecond laser ablation of a NMP aqueous solution (which comprises nitrogen-containing organic solvents) causes nitrogen-containing functional groups to be adsorbed on the surface of the as-prepared MoS_2_ QDs. This may result in a substantial enhancement of PL, as was observed elsewhere in GQDs^51^.

**Figure 7:**
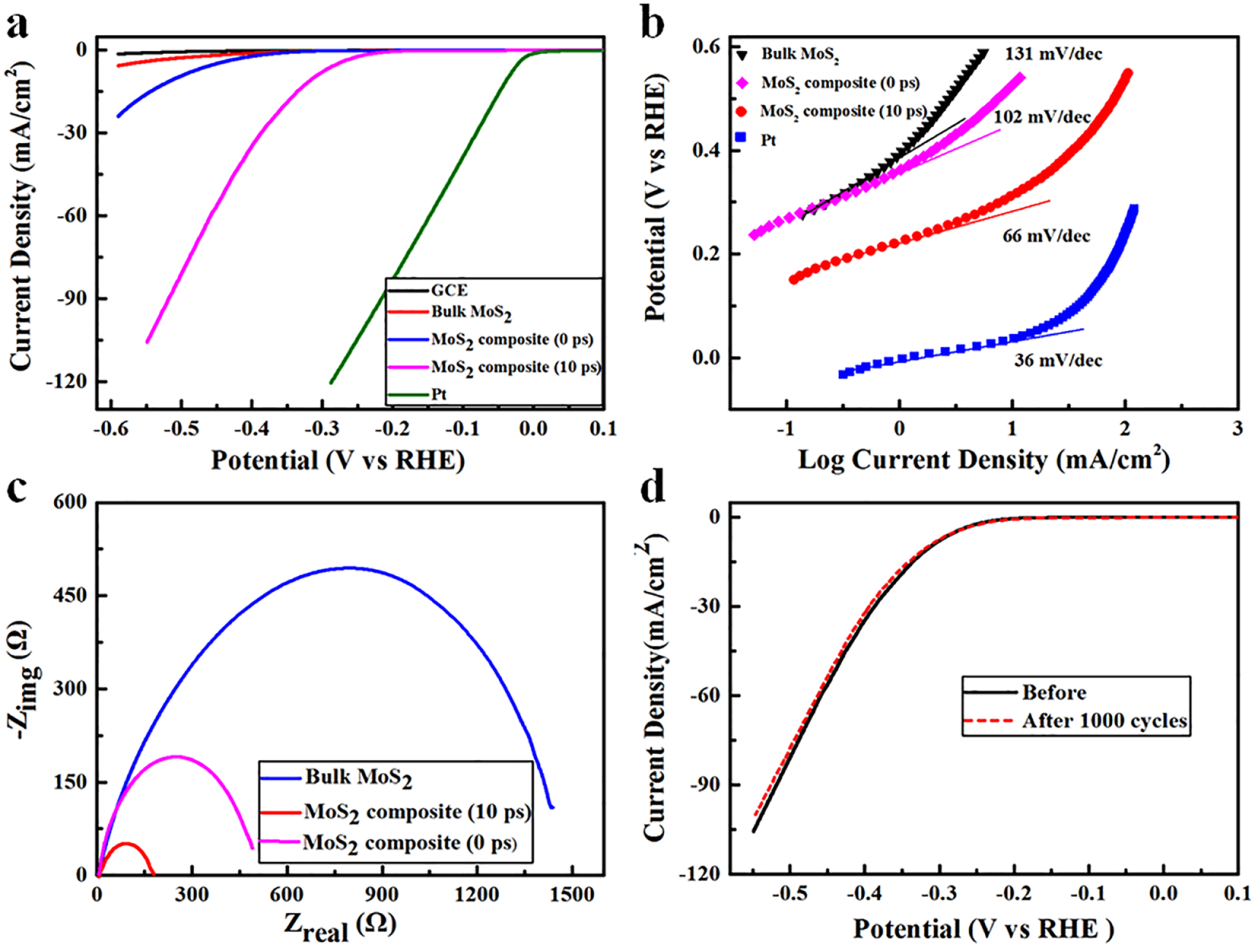
HERs activity of the as-prepared MoS_2_ materials. (**a**) Polarization curves. (**b**) Corresponding Tafel plots of bulk MoS_2_, MoS_2_ composites (0 ps), MoS_2_ composites (10 ps), and Pt electrodes at a scan rate of 5 mV s^−1^ in 0.5 M H_2_SO_4_. (**c**) EIS Nyquist plots of bulk MoS_2_, MoS_2_ composites (0 ps), and MoS_2_ composites (10 ps). (**d**) Stability test of MoS_2_ composites (10 ps).

The as-prepared MoS_2_ materials were used as efficient electrocatalysts for HERs. The HERs activity of these materials was explored in an N_2_-saturated 0.5 M H_2_SO_4_ electrolyte in a typical three-electrode electrochemical workstation by using a standard rotating-disk glassy carbon electrode (GCE, 5 mm diameter) as the working electrode. A Pt wire electrode and a Ag/AgCl electrode were used as the counter and reference electrodes, respectively. The GCE was modified using MoS_2_ composites (10 ps) (composite of nanosheets, nanoparticles, and QDs prepared using temporally shaped femtosecond laser) for linear sweep voltammetry measurements at a scan rate of 5 mV s^−1^. For comparison, similar measurements were also conducted on a bare GCE, a GCE modified using bulk MoS_2_, a GCE modified using MoS_2_ composites (0 ps) (composite of nanosheets, nanoparticles, and QDs prepared using femtosecond laser single pulse), and a commercial Pt electrode (20 wt% Pt/C). The obtained polarization curve of the Pt catalyst displays extremely high HERs catalytic activity with a near-zero onset overpotential (Fig. 7a). However, the bare GCE and bulk MoS_2_ exhibit extremely low HERs catalytic activity with a high onset overpotential of approximately 350 and 320 mV, respectively. Moreover, their cathodic current density is very weak, hardly increasing with the negative potential. By contrast, the MoS_2_ composites (0 ps) show a slightly lower onset overpotential of approximately 260 mV. While the MoS_2_ composites (10 ps) exhibit a relatively high HERs catalytic activity with a low onset overpotential of approximately 140 mV, and the cathodic current density increases rapidly with an increase in the negative overpotential. At approximately 400 mV, the as-prepared MoS_2_ composites (10 ps) display an extremely large cathodic current density of 36 mA cm^−2^. Whereas the MoS_2_ composites (0 ps) display a cathodic current density of 2.2 mA cm^−2^. Notably, the HERs catalytic activity of the as-prepared MoS_2_ composites (10 ps) is comparable with that in other recent reports^21, 22^.

The Tafel slope is another key parameter in evaluating the HERs catalytic activity of catalysts; it reflects the intrinsic property of the electrocatalytic materials and is determined by the rate-limiting step of the HERs. The smaller the Tafel slope is, the faster is the hydrogen generation rate obtained at the applied negative overpotential. The Tafel slope is obtained by fitting the linear portions of the Tafel plots to the Tafel equation (*η* = *b log* |*j*| + *a*, where *j* is the current density and *b* is the Tafel slope). As shown in Fig. 7b, the Tafel plots derived from the polarization curves indicate that the commercial Pt has a Tafel slope of approximately 36 mV dec^−1^, which is close to that identified by other previous reports^18, 21, 52^. For MoS_2_ composites (10 ps), the slope is approximately 66 mV dec^−1^, which is much smaller than that of the MoS_2_ composites (0 ps) (approximately 102 mV dec^−1^) and bulk MoS_2_ (approximately 131 mV dec^−1^). This demonstrates that the HERs catalytic activity is determined by the Volmer-Heyrovsky reaction^9^. Considerable improvement was seen between bulk MoS_2_ and MoS_2_ composites (10 ps). Indeed, the Tafel slope of the MoS_2_ composites prepared through temporally shaped femtosecond laser two-subpulse train ablation is comparable to or smaller than that of the composites prepared through either a hydrothermal approach^21^, sonication combined with solvothermal treatment synthesis^18^, or electrochemical etching^22^. Nevertheless, the slope is poorer than that of 1 T phase MoS_2_ nanosheets prepared through chemical exfoliation^12, 53, 54^ or MoS_2_ heterostructures on reduced graphene oxide^55^. A comparison of the HERs catalytic activity of the MoS_2_ composites prepared using a femtosecond laser with the MoS_2_-based HERs catalysts prepared using other typical synthesis methods is presented in Supplementary Table S1.

Compared with the MoS_2_ composites prepared using a femtosecond laser single pulse, an obvious improvement in the HERs catalytic activity can be obtained using MoS_2_ composites prepared by a temporally shaped femtosecond laser. This may be attributed to several reasons. First, there is a much higher ratio of small-size QDs prepared and a reduction of the lateral and vertical dimensions to monolayer ultrasmall QDs when suing a temporally shaped femtosecond laser (see the TEM images in Fig. 3 and Figure S3). This produces more available edge sites for hydrogen adsorption. Second, there is a much higher production rate of MoS_2_ nanomaterials prepared that can be estimated from the concentration of the as-prepared MoS_2_ aqueous solutions (see Fig. 2) when using a temporally shaped femtosecond laser. Finally, more MoS_2_ materials were thinned to monolayer zero-dimensional QDs when using a temporally shaped femtosecond laser, which resulted in a high edge-to-basal plane ratio. Notably, this results in considerable improvement of the carrier mobility, which further enhances the efficiency of electron transfer between the active edge sites and the underlying electrode^18, 56^; and is revealed through electrochemical impedance spectroscopy. As shown in Fig. 7c, the Nyquist plots demonstrate that the electron transfer resistance considerably reduces from bulk MoS_2_ (1500 Ω) to MoS_2_ composites (0 ps) (500 Ω) and MoS_2_ composites (10 ps) (180 Ω), resulting in a faster electron transfer between the MoS_2_ composite catalysts and the electrode.

Finally, we measured the stability and durability of the MoS_2_ composite (10 ps) catalysts under continuous operation. As shown in Fig. 7d, the HERs catalytic activity of these MoS_2_ composites (10 ps) had no obvious change even after 1000 continuous cycles. This stability is comparable to that reported in prior research^9, 44, 45, 52, 57, 58^. Moreover, there is only an approximately 20% loss of cathodic current when a potential of −300 mV *vs*. reversible hydrogen electrode (RHE) is applied for 5000 seconds of continuous operation (Supplementary Figure S8), an outcome which reflects results elsewhere^21^.

## Conclusion

In conclusion, we reported a novel, fast, green, and one-step approach to prepare monolayer MoS_2_ QDs using the temporally shaped femtosecond laser ablation of bulk MoS_2_ targets in water. We achieved a uniform size distribution monolayer and a high production rate of MoS_2_ QDs by simply adjusting the laser power and the two-subpulse delay within a pulse train. The as-prepared MoS_2_ QDs possess abundant active edge sites, high carrier mobility, and a large specific surface area; consequently, the QDs exhibit excellent electrocatalytic activity for the HERs with an onset overpotential of approximately 140 mV, a Tafel slope of approximately 66 mV dec^**−**1^, long-term durability, and high stability. In addition, MoS_2_ QDs possess strong quantum confinement and edge effects that, when combined with the direct bandgap property, afford them with unique PL features. Thus, MoS_2_ QDs have great potential in the biomedical and optical-imaging applications. Furthermore, the production rate of MoS_2_ nanomaterials might be further enhanced through multi-beam parallel processing or optimization of other laser parameters, such as scan speed, laser wavelength, and pulse delay, *etc*. In short, this study provided a novel, simple, green, and one-step approach to facilely and efficiently produce QDs of TMDs or other layered materials for broad potential applications.

## Methods

### Materials

Original bulk 2H-MoS_2_ and WS_2_ targets (10 × 10 × 5 mm^3^) were purchased from Jiangxi Ketai Advanced Materials Co. Ltd, Jiangxi, China. Original GO dispersions were purchased from Nanjing XFNANO Materials TECH Co.,Ltd, China. NMP and H_2_SO_4_ (98%) were acquired from Sinopharm Chemical Reagent Co. Ltd. Nafion solution (5%) was purchased from Dupont China Holding Co. Ltd. N_2_ with a purity of 99.9% was purchased from Beijing Hairui Tongda Gas Technology Co. Ltd. All reagents were of analytical grade and used without further purification.

### Preparation of MoS_2_ Quantum Dots

An amplified Ti:sapphire laser system (Spectra Physics Inc.) was used to generate linearly polarized laser pulses of 50 fs (FWHM) pulse duration at 800 nm central wavelength with a repetition rate of 1 kHz. Collinear two-subpulse irradiation was obtained using a Michelson interferometer, which splits each pulse into two temporally separated subpulses with nearly identical pulse characters. The temporal separation (τ_s_), which ranges from 100 fs to tens of picoseconds, was adjusted using a computer-controlled linear translation stage with a micrometer resolution. Before laser ablation, the bulk MoS_2_ targets were dipped into ethanol and then water for ultrasonic cleaning for 10 minutes using a sonicator (KQ3200DB). Then, the bulk MoS_2_ targets were placed at the bottom of a glass vessel filled with 5 mL of distilled water. The typical thickness of the liquid above the targets were approximately 3 mm. The glass vessel was mounted on a computer-controlled six-axis positioning system (M-840.5DG, PI, Inc.), and the targets were continuously moved during laser irradiation to obtain a fresh surface for ablation. The laser beam was focused normally on the surface of the target by using a plano-convex lens (f = 100 mm). The MoS_2_ QDs were prepared using a temporally shaped femtosecond laser two-subpulse train with a total energy of 3.5 μJ (0.77 J cm^−2^), and the intensity of the two subpulses was approximately 1:1. The pulse delay between the two subpulses was 10 ps. When using a conventional femtosecond laser single pulse, the pulse delay between the two subpulses was 0 ps. The pulse energy was finely controlled using a half-wave plate combined with a polarizer and was mounted prior to the interferometer entrance. The preparation was performed in air at ambient pressure and temperature. After ablation for 2 hours, the MoS_2_ aqueous solution was allowed to settle for 2–4 hours and was then centrifuged at 8000 rpm for 10 minutes to obtain the transparent light-yellow supernatant solution. Subsequently, the supernatant solution was sonicated for 1 hour in an ice bath to avoid the agglomeration of QDs, which can be directly applied for further characterization. For HERs, we used MoS_2_ composites that comprised MoS_2_ nanosheets, nanoparticles, and QDs before centrifugation. In addition to water, the proposed methodology can be applied to other aqueous media such as NMP; the other experimental parameters and procedures are the same as those applied in the preparation of QDs in water. The WS_2_ QDs were prepared using the same experimental setup and process as that for the preparation of MoS_2_ QDs. However, the pulse energy and pulse delay were changed to 2 μJ (0.44 J cm^−2^) and 2 ps, respectively. The concentration of the original GO dispersions was 2 mg/mL, which we diluted to 0.5 mg/mL using deionised water. The total power (density) of the temporally shaped femtosecond laser pulse two-subpulse train was 200 mW (44 J/cm^2^) and the pulse delay between the two subpulses was 10 ps. After 1 hour of femtosecond laser two-subpulse train ablation of the GO dispersions, we obtained GQDs.

### Electrochemical Measurements

The HERs catalytic activity of the as-prepared MoS_2_ composites (composite of nanosheets and QDs) was evaluated using a computer-controlled electrochemical workstation (CHI 760D) in a standard three-electrode system. The rotating-disc GCE was used as the working electrode while a Ag/AgCl (in 3.5 M KCl solution) electrode was used as the reference electrode; a Pt wire was used as the counter electrode. The MoS_2_ composites working electrode was prepared as follows: (1) 10 μL of MoS_2_ composites solution was dropped onto a GCE with 5 mm diameter using a pipettor and dried at room temperature; (2) the process of (1) was repeated five times; (3) 5 μL of Nafion solution (5%) was coated on the GCE. All the measurements were performed in 0.5 M H_2_SO_4_ solution. Before the experiments, the electrolyte was degassed in bubbling N_2_ for 30 minutes. Subsequently, the GCE was pre-conditioned (prior to collecting polarization curves) through cyclic voltammetry (CV) at a scan rate of 100 mV s^−1^ for 200 cycles while the GCE was rotated at 1600 rpm. After the CV, linear sweep voltammetry (LSV) was performed at a scan rate of 5 mV s^−1^ while the working electrode was rotated at 1600 rpm. iR compensation was performed for all the polarization curves of all catalysts. All the potentials obtained in our study were through RHE calibration against the RHE. Electrochemical impedance spectroscopy (EIS) was performed using the same configuration at an overpotential *η* = −0.35 V vs RHE from 10^6^ to 1 Hz at an alternating current voltage of 5 mV. The stability and durability of the MoS_2_ composites were evaluated through CV for 1000 cycles with a scan rate of 100 mV s^−1^ and continuous operation of 5000 s at an overpotential *η* = −0.3 V *vs*. RHE while rotating the working electrode at 1600 rpm, respectively.

### Characterization

The SEM images were obtained using a field emission environmental scanning electron microscope (QUANTA 200 FEG). The concentration of MoS_2_ aqueous solutions were characterized using an optical emission spectroscope with inductively coupled plasma (Varian Vista-MPX). The TEM images were captured with a JEM2010 at an accelerating voltage of 200 KV. AFM images were obtained using a Bruker Multimode 8 in the tapping mode after the samples were deposited on a freshly cleaved mica surface through the drop-casting method. XRD data were obtained using D8 ADVANCE (Bruker) with Cu–Kα radiation (40 kV, 40 mA). Raman spectra were acquired through inVia-reflex microconfocal laser Raman spectroscopy (Renishaw) with the excitation laser line at 633 nm. XPS analysis was performed on a ESCALAB 250Xi spectrometer (Thermo Fisher) with an Al Kα X-ray source. UV–vis spectra were obtained using a U-3900 spectrophotometer. Fluorescence spectroscopy was performed using a F-7000 FL spectrophotometer.

## Electronic supplementary material

Supplementary Information
