# Supplementary material for: Preparation of Monolayer MoS2 Quantum Dots using Temporally Shaped Femtosecond Laser Ablation of Bulk MoS2 Targets in Water
Source: Sci Rep. 2017 Sep 11;7:11182. doi: 10.1038/s41598-017-10632-3 (PMC5593836; doi:10.1038/s41598-017-10632-3)
Supplement: Supplementary file 1 — Supplementary Information [file 41598_2017_10632_MOESM1_ESM.pdf]

Supplementary Information

Preparation of Monolayer MoS_2 Quantum Dots using Temporally Shaped Femtosecond Laser Ablation of Bulk MoS_2 Targets in Water

Bo Li[1], Lan Jiang[1,*], Xin Li[1], Peng Ran[1], Pei Zuo[1], Andong Wang[1], Liangti Qu[2], Yang Zhao[2], Zhihua Cheng[2], Yongfeng Lu[3]

[1]Laser Micro/Nano Fabrication Laboratory, School of Mechanical Engineering, Beijing Institute of Technology, Beijing, 100081, PR China

[2]Key Laboratory of Cluster Science, Ministry of Education, School of Chemistry, Beijing Institute of Technology, Beijing 100081, PR China

[3]Department of Electrical and Computer Engineering, University of Nebraska-Lincoln, Lincoln, NE 68588-0511, USA.

[*]*jianglan@bit.edu.cn*

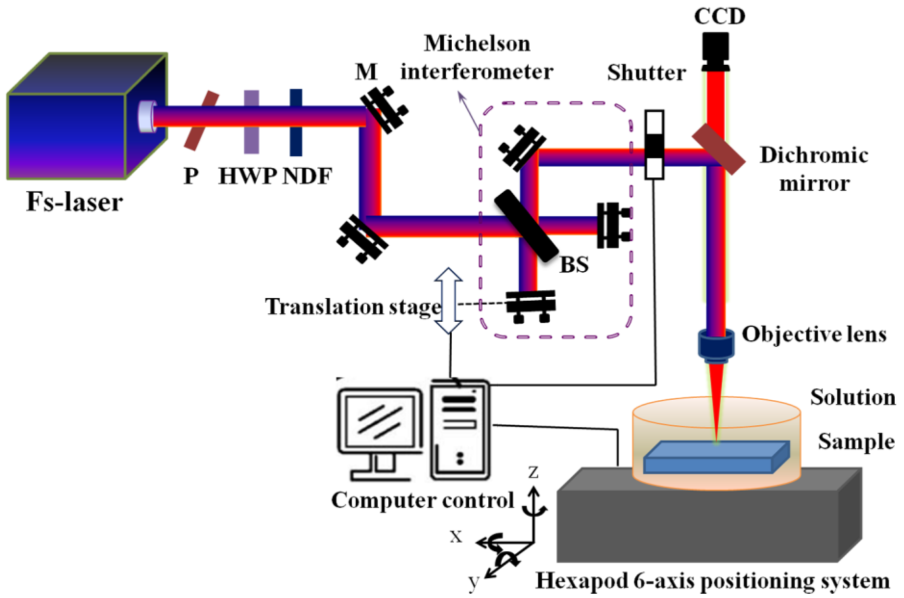

Figure S1. Schematic of the experimental setup for two-subpulse generation. P: polarizer; HWP: half-wave plate; NDF: neutral density filters; M: mirror; BS: beam splitter; CCD: charge coupled device.

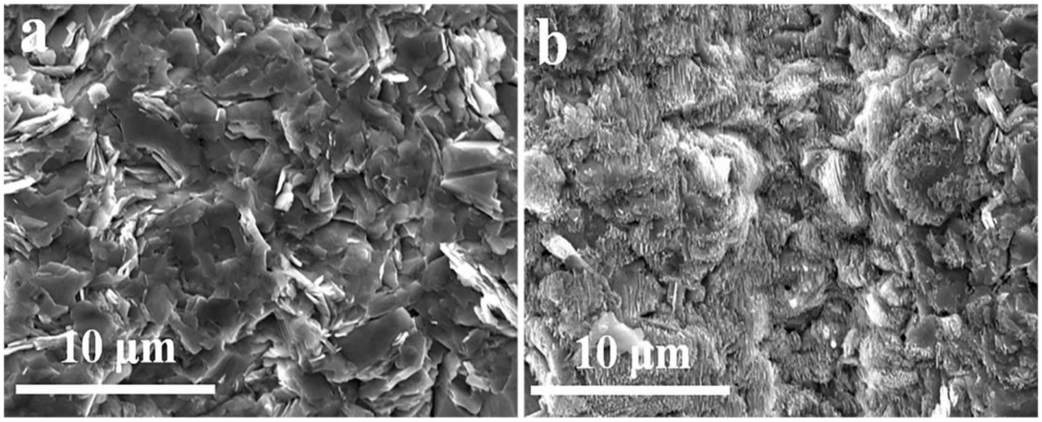

Figure S2. SEM images of the surface of original bulk MoS_2 target (a) before and (b) after femtosecond-laser temporally shaped two-subpulse train ablation in water.

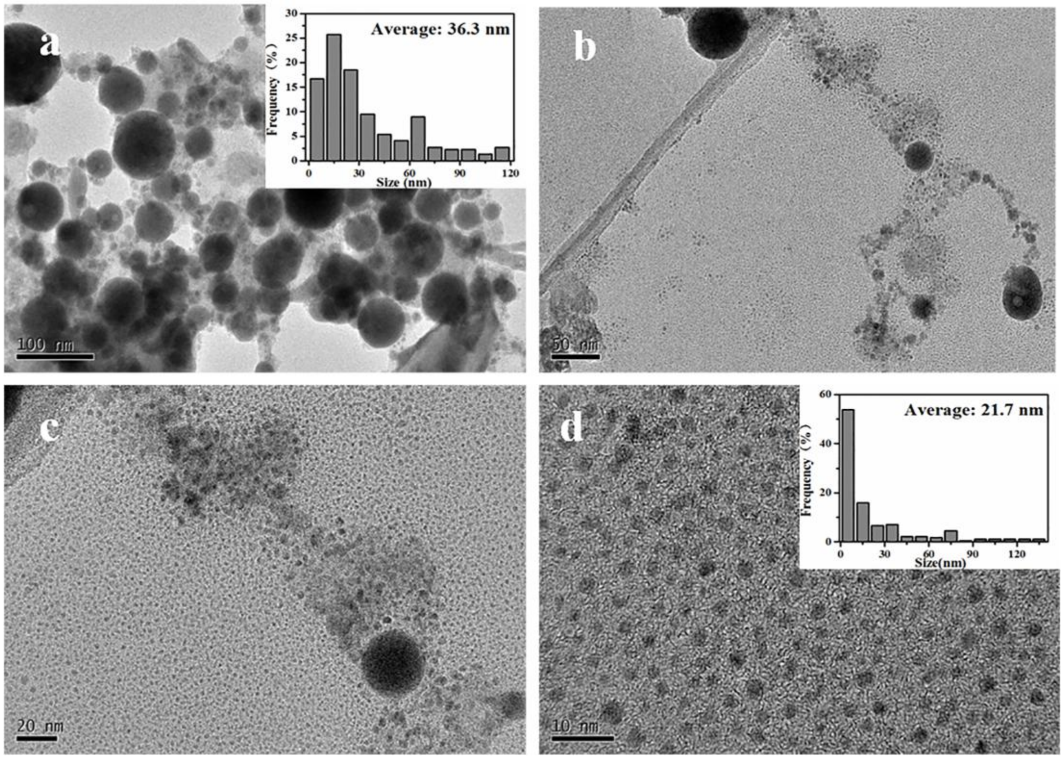

Figure S3. The TEM images corresponding to the morphology of MoS_2 nanomaterials in the aqueous solutions before centrifugation: (a) obtained by femtosecond laser single pulse ablation; (b), (c), and (d) obtained by temporally shaped femtosecond laser two-subpulse train ablation. The insert image of a and d show the size distribution. The scar bars are 100 nm, 50 nm, 20 nm, and 10 nm, respectively.

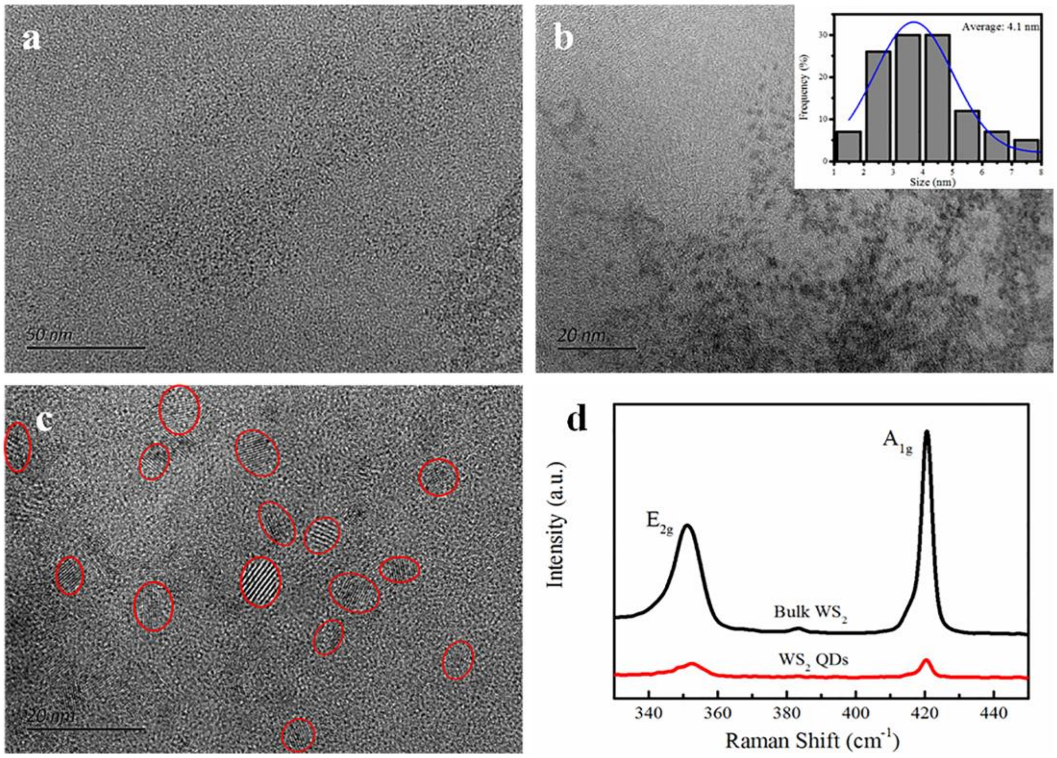

Figure S4. (a-c) TEM and HRTEM images of the as-prepared WS_2 QDs, the scale bars are 50 nm, 20 nm, and 20 nm, respectively. The inset in (b) is the size distribution of the WS_2 QDs. (d) The Raman spectrum of the bulk WS_2 and WS_2 QDs.

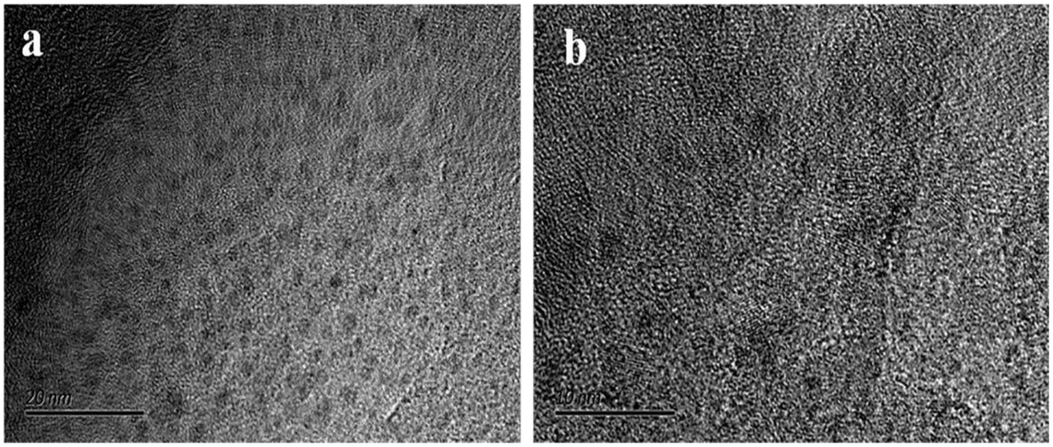

Figure S5. The TEM image of the as-prepared GQDs. The scale bars are 20nm and 10 nm, respectively.

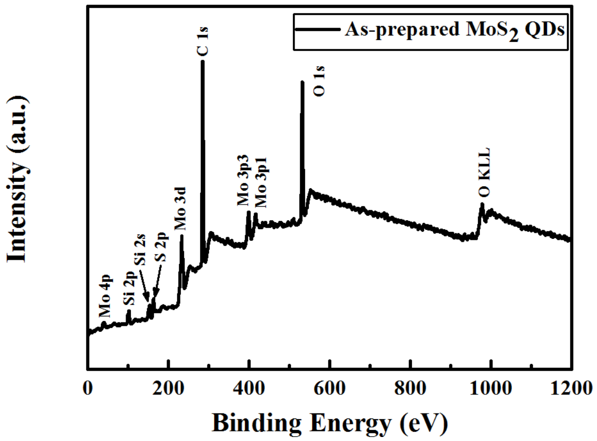

Figure S6. The full spectrum of XPS survey of the as-prepared MoS_2 QDs.

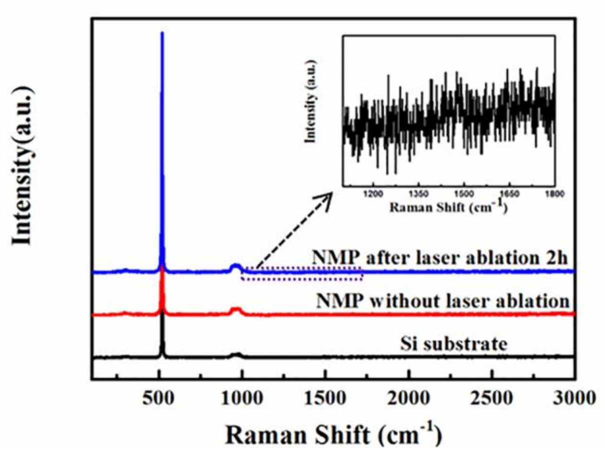

Figure S7. The Raman spectra of the Si substrate, NMP solution without laser ablation, and NMP solution after laser ablation for 2h, respectively. And the inset image is the local magnification of the spectra of NMP solution after laser ablation for 2h.

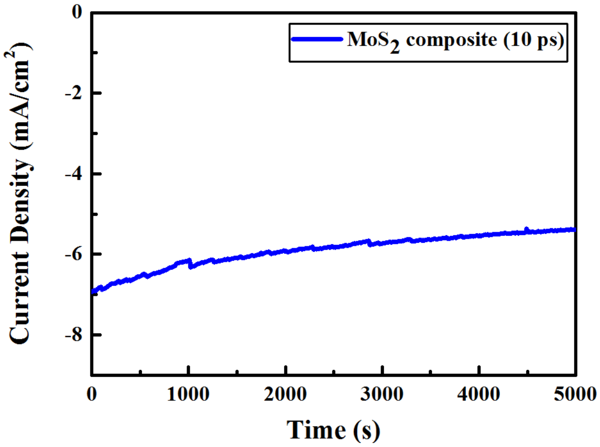

Figure S8. The durability test of MoS_2 composites (composite of MoS_2 nanosheets, nanoparticles, and QDs obtained by temporally shaped femtosecond laser before centrifugation) with an applied voltage of -0.3 V vs RHE over 5000 seconds in 0.5 M H_2SO_4.

Table S1. Comparison of HERs performance with the MoS_2-based HERs catalysts prepared by other typical synthesis methods.

**Synthesis methods**	**Catalysts**	**Onset potential (V)**	**Overpotential (V)**	**Tafel slope (mV dec^{-1})**	**Refs.**
**Precursors reaction**	Defect-free MoS_2 NSs	-0.18	/	87	56
**Sonication combined with centrifugation**	MoS_2 NPs	-0.16	-0.2 (0.4 mA cm^{-2})	82	S2
**Liquid exfoliation**	MoS_2 QDs on NSs	-0.19	-0.4 (120 mA cm^{-2})	74	20
**Sonication combined with solvothermal**	MoS_2 QDs	-0.12	/	69	18
**Ultrasonication combined with centrifugation**	MoS_2 NPs	-0.09	-0.15 (0.92 mA cm^{-2})	69	45

**Ionic liquid assisted grinding exfoliation**	MoS_2 NDs	-0.09	-0.248 (10 mA cm^{-2})	61	9
**Electrochemical etching**	MoS_2 QDs	-0.21	/	60	22
**Hydrothermal**	MoS_2 QDs	-0.16	-0.4 (39 mA cm^{-2})	59	21
**Precursors reaction**	Defect-rich MoS_2 NSs	-0.12	-0.12 (13 mA cm^{-2})	50	56
**Chemical exfoliation**	MoS_2 NSs on graphite	/	-0.187 (10 mA cm^{-2})	43	53
**Solvothermal**	MoS_2 NPs on RGO	-0.1	/	41	S1
**Temporally shaped femtosecond-laser ablation**	MoS_2 composites	-0.14	-0.4 (36 mA cm^{-2})	66	This work

NSs: Nanosheets

NPs: Nanoparticles

NDs: Nanodots

RGO: Reduction graphene oxide

References

S1. Y. Li, H. Wang, L. Xie, Y. Liang, G. Hong and H. Dai, *J. Am. Chem. Soc.* **2011**, *133*, 7296.

S2. T. Y. Wang, D. L. Gao, J. Q. Zhuo, Z. W. Zhu, P. Papakonstantinou, Y. Li, M. X. Li, *Chem.–Eur. J.* **2013**, *19*, 11939.
